# Whether Modulating the Activity of the Temporalparietal Junction Alters Distribution Decisions within Different Contexts: Evidence from a tDCS Study

**DOI:** 10.3389/fpsyg.2017.00224

**Published:** 2017-02-21

**Authors:** Jun Luo, Shu Chen, Daqiang Huang, Hang Ye, Haoli Zheng

**Affiliations:** ^1^Neuro & Behavior EconLab, School of Economics, Center for Economic Behavior and Decision-Making, Zhejiang University of Finance and EconomicsHangzhou, China; ^2^School of Economics and Interdisciplinary Center for Social Sciences, Zhejiang UniversityHangzhou, China

**Keywords:** distributive decision, social preference, advantage inequity aversion, temporoparietal junction, transcranial direct current stimulation

## Abstract

Distributive justice concerns how individuals and societies distribute income in a just or equal manner. We aimed to test the roles of social preference in behavioral distributive justice. We thus provide evidence of a causal link between the neural and behavioral results through the application of bilateral transcranial direct current stimulation (tDCS) over the temporoparietal junction (TPJ) of our participants. The participants were found to make fairer distributions within the known position after receiving right anodal/left cathodal tDCS and receiving right cathodal/left anodal tDCS over the TPJ than the participants who received the sham stimulation. Simultaneously, we elicited the participants’ advantage inequity aversion and found that the participants who received right anodal/left cathodal tDCS and who received right cathodal/left anodal tDCS over the TPJ were more averse to advantage inequity. Additionally, the participants’ distributive proportions to the lowest income stratum within the known position were strongly related to their social preference of advantage inequity aversion. Therefore, the present study demonstrated that the modulation of the excitability of the TPJ using tDCS altered the distributive decisions of the participants within the known position, and this effect might be attributable to a change in the individuals’ social preferences.

## Introduction

The issues surrounding economic fairness and distributive justice arise in the context of allocation problems and focus on the normative question of how the allocation should be performed. Formally, distributive justice is concerned with “what rules, procedures, or mechanisms a society or group should use to allocate its scarce resources, commodities, and necessary burdens among individuals with competing needs and claims.” There are many empirical studies of issues that concern how a society or group allocates benefits or burdens in a just or moral manner through the use of three different approaches.

The veil of ignorance approach describes a decision making environment in which hypothetical rational individuals make decisions from an “original position” prior to entering society, without any knowledge of what their position in society might be or what individual attributes or circumstances they will face (Vickrey, 1945; Harsanyi, 1955; Rawls, 1971). Therefore, choices made in the “original position” behind a “veil of ignorance” are made in a state of uncertainty. The goal of using the “veil” of imperfect information is to strip away any prejudices from history, status quo property rights, and institutions so that impartial decisions based on the formal principle of distributive justice can be made.

The social-planner approach assumes that some outside judge or observer, a social planner, or some impersonal social welfare function, evaluates the equity of income distribution. The approach lacks any personal involvement because the social-planner does not become a member of the society (Brandt and Boulding, 1962; Cowell and Kuga, 1981; Lambert, 2001; Traub et al., 2005, 2009). This procedure required him or her to genuinely behave as an umpire for income distribution. The social-planner compiled their orderings of income sets without having any stakes in the outcomes. The umpire himself or herself was excluded from any chance of receiving a payoff.

The known position approach sets an authority, leader, or member of the highest income stratum to make decisions for income distribution. They know their prominent roles in this society before they make decisions. They had to determine both their own payoffs and the payoffs of their society members. He or she was called to the forefront and became visible to all other members to strengthen his or her social responsibility in the face of the whole public (Sutter and Weck-Hannemann, 2003; Engelmann and Strobel, 2004; Traub et al., 2009). This approach establishes that a person’s social preference in terms of maximizing a social welfare function of the payoff distribution is mixed with his or her selfish motive in terms of maximizing his or her own payoff.

In the veil of ignorance approach, income distributions share a striking similarity with lotteries (Friedman, 1953; Dahlby, 1987). However, for the evaluation of income distribution, it is often argued that individuals develop social preference and would thus, in contrast to lotteries, exhibit both a risk component and an altruism component in their behavior (Cowell and Schokkaert, 2001). The social-planner approach assumes that the planner maximizes the social welfare of an external society, and the lack of personal involvement within this context appears to induce a moderate degree of inequality aversion (Traub et al., 2005). Under the known position approach, the authority’s preferences determined the choice of the prevailing income distribution. The utility of a person is assumed to depend not only on his or her own monetary payoff but also on a specific social welfare function of the payoff distribution. Hence, results that were obtained on the known position approach support recent experimental evidence on social preferences (Fehr and Schmidt, 1999; Bolton and Ockenfels, 2000; Andreoni and Miller, 2002; Charness and Rabin, 2002).

Most of the above studies used non-incentivized questionnaires that ask participants to choose between lotteries representing different income distributions from the perspective of an uninvolved outside observer, i.e., from behind the veil of ignorance and a purely individual risk perspective (Bernasconi, 2002; Amiel et al., 2009). In this study, we elicit preferences over income distribution in an incentive compatible manner and test how such preferences relate to some simple notions of income justice. We focused on “fixed pie” type problems in which the initial endowment of items is to be distributed into three different social stratums, and the participants are required to have a size order for the distributive income across different stratums.

To test the role of social preference in behavioral distributive justice, we utilized a controlled laboratory setting with three different distributive contexts, which included a veil of ignorance, a social-planner and a known position. In the first distributive context, the participants do not know which future position in society they (as well as other individuals) will be assigned when deciding how to distribute the initial endowment across the different stratums. In the second distributive context, participants will not be assigned a future position in society and will receive a fixed payoff as a social-planner when deciding how to distribute the initial endowment across the different stratums. In the third distributive context, the participants know that they will be assigned to the richest stratum in society when they decide how to distribute the initial endowment across the different stratums. In addition, we added a choice menu to measure participants’ advantage inequity aversion in the experiment.

Despite the long history of work on distributive behavior, its psychological and neural underpinnings remain poorly understood, and much of the work has centered on the intentions of decisions. The previous studies include many debates on whether and how the “weights” that are assigned to the individual payoffs (self-interest or risk aversion) and the payoff distributions of the others (social preference or inequity aversion) in the participants’ objective functions.

Essentially, there are obvious distinctions between the two different distributive intentions in neural substrates. Previous clinical and neuroimaging studies have revealed the involvement of a distributed bihemispheric, corticosubcortical network in decision making (Ernst and Paulus, 2005; Krain et al., 2006). The dorsolateral prefrontal cortex (DLPFC) is an important part of this network (Manes et al., 2002; Clark et al., 2003) and appears to be particularly involved in decision making when choices are ambiguous (Krain et al., 2006). This connection is of particular relevance in light of the growing evidence that this region is involved in risky decisions (Kuhnen and Knutson, 2005; Preuschoff et al., 2006; Cazzell et al., 2012; Holper et al., 2014).

In contrast, a wide variety of neuroimaging studies can provide positive evidence to support the hypothesis that altruism derives, at least in part, from the tendency to consider others’ states, and experiments with adults indicate that subjects with better skills in reading others’ states show more altruistic behavior (Underwood and Moore, 1982). One brain region that has been repeatedly and reliably found to be implicated in tasks that require the ability to represent and understand others’ perspectives is the temporoparietal junction (TPJ) (Ruby and Decety, 2001; Saxe and Kanwisher, 2003; Decety and Lamm, 2007; Frith and Frith, 2007; Young and Dodell, 2010). If altruistic behavior is indeed supported by an appreciation of others’ perspectives, then the TPJ should play an important role in decisions to act altruistically.

Neuroimaging studies are useful for establishing correlations between brain activations and processes of considering others’ perspectives (social preference), but they do not provide information regarding whether a given region is necessary to the resulting behavior. Non-invasive brain stimulation techniques, such as repetitive transcranial magnetic stimulation (rTMS), transcranial direct current stimulation (tDCS), allow for the study of the behavioral consequences of an externally induced brain activation or inactivation in healthy participants and thus enable the establishment of a causal relationship between the TPJ and social preference (Knoch et al., 2006; Fecteau et al., 2007a,b; Ye et al., 2015a).

The main objective of the present paper was to provide neural evidence for intrinsic preference in different contexts of income distribution and to test whether distributive decisions in different contexts are driven by social preference. We performed an income distribution experiment to investigate whether bilateral stimulation of the TPJ (anodal stimulation of the right and cathodal stimulation of the left TPJ or vice versa) would alter distributive decisions in different contexts. By comparing the values of advantage inequity aversion in the choice menu across different tDCS stimulations, a causal relationship between the excitability of the TPJ and social preference might be observed. Based on these results, we can infer that the modulation of the activity of the TPJ might alter the distributive decisions that are made within the known position through their main driving force, i.e., individual advantage inequity aversion.

## Materials and Methods

### Subjects

We recruited 78 healthy college students (39 females; mean age 19.3 years, ranging from 17 to 25 years) to participate in our experiment. All participants were right-handed and naïve to tDCS and distributive tasks, with no history of psychiatric illness or neurological disorders. The participants were randomly assigned to receive right anodal/left cathodal tDCS (*n* = 26, 13 females), left anodal/right cathodal tDCS (*n* = 26, 13 females) or sham stimulation (*n* = 26, 13 females). The final payoff was a fixed show-up fee of 20 RMB Yuan (approximately 3 US dollars) plus the reward gained from the distributive tasks. The participants received 52.5 RMB Yuan (approximately 7.9 US dollars) on average, fluctuating according to their performance. Participants gave informed written consent before entering the study, which was approved by the Zhejiang University ethics committee. No participants reported any adverse side effects concerning pain on the scalp or headaches after the experiment.

### Transcranial Direct Current Stimulation

Transcranial direct current stimulation applied a weak direct current to the scalp via two saline-soaked surface sponge electrodes (35 cm^2^). The current was constant and delivered by a battery-driven stimulator (Starlab, Spain), which was controlled through a Bluetooth signal. It was adjusted to induce cortical excitability of the target area without any physiological damage to the participants. Various orientations of the current had various effects on the cortical excitability. Generally speaking, anodal stimulation enhances cortical excitability, whereas cathodal stimulation restrains it (Nitsche and Paulus, 2000).

**FIGURE 1:**
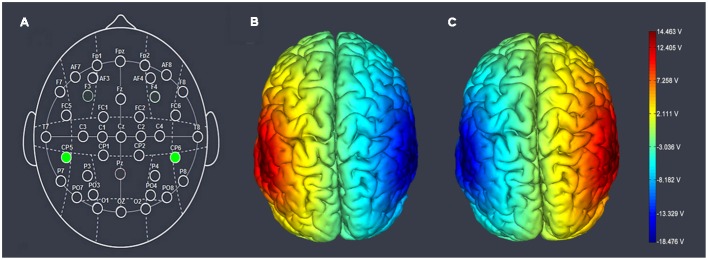
**Schematic drawing of electrode positions suited for tDCS of the temporoparietal junction of the human brain. (A)** Stimulation of the respective cortices according to the 10–20 system. **(B)** The electrode placement of left anodal/right cathodal stimulation. The anodal electrode was placed over CP5 and the cathodal electrode was placed over CP6. **(C)** The electrode placement of left cathodal/right anodal stimulation. The anodal electrode was placed over CP6 and the cathodal electrode was placed over CP5. The axis represents the range of input voltage from -18.476v to 14.463v.

Participants were randomly assigned to one of three treatments. For right anodal/left cathodal stimulation, the anodal electrode was placed over the right CP6 according to the international EEG 10–20 system, while the cathodal electrode was placed over the left CP5. For left anodal/right cathodal stimulation the placement was reversed. The anodal electrode was placed over CP5 and the cathodal electrode was placed over CP6 (**Figures 1A–C**). For sham stimulation, the procedures were the same but the current lasted only for the first 30 s. The participants may have felt the initial itching, but there was actually no current for the rest of the stimulation. This method of sham stimulation has been shown to be reliable (Gandiga et al., 2006). The current was constant and of 2 mA intensity with 15 s of ramp up and down, the safety and efficiency of which was shown in previous studies (Jacobson et al., 2012).

Before the decision making tasks, the laboratory assistant put a tDCS device on the participant’s head for stimulation. After 20 min of stimulation, the tDCS device was taken off and the participant was then asked to complete distributive decision task and a choice menu. It has been demonstrated that bilateral tDCS over cortical brain regions facilitates greater improvements for healthy subjects’ compared to uni-lateral stimulation (Vines et al., 2008; Williams et al., 2010; Sehm et al., 2013). That is the reason we chose a bifrontal electrode montage was to provide stimulation able to enhance the activity of one side of the TPJ while simultaneously diminishing the other side (see Sellaro et al., 2016 for further discussing of the bilateral stimulation).

### Task and Procedure

After the participants received tDCS stimulation for 20 min (bilateral stimulation, single-blinded, sham-controlled), they completed an income distributive task (the computer program for this task was written in visual C#).

**FIGURE 2:**
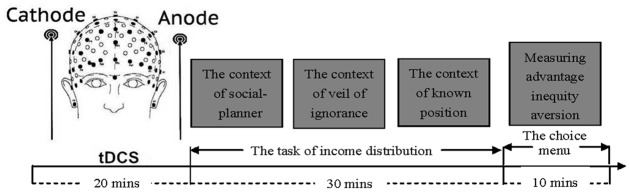
**Schematic representation of the experimental design.** After 20 min of stimulation, each participant was then asked to complete the distribution task in the three types of distributive context and the choice menu.

The task consists of 30 stories, and each story includes a distributive context and a question about how to distribute an initial endowment among three stratums (**Figure 2**). These stories involve three types of distributive context (social-planner, the veil of ignorance and known position) with 10 levels of initial endowments (30, 60, 90, 120, 150, 180, 210, 240, 270, 300 chips), and 50 chips = 1 RMB Yuan. The participants could choose freely which amounts to give each of the three stratums in this task.

**FIGURE 3:**
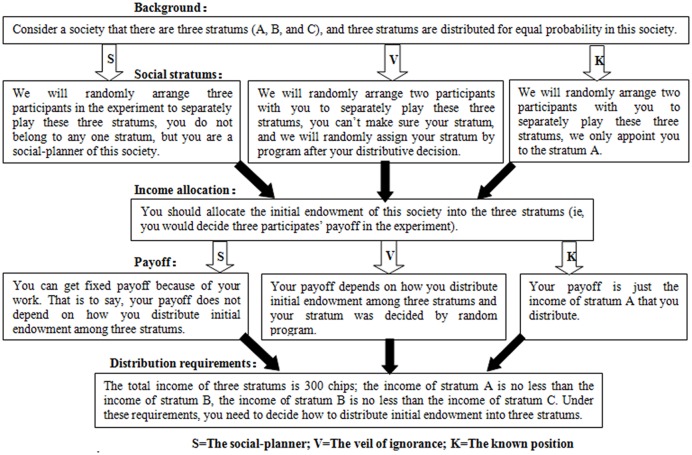
**A story within the context of social-planner, veil of ignorance and known position presenting on the computer screen**.

To avoid the order effect and income effect, we assigned three fixed orders (pseudo-random order) in which all stories were presented on the screen, and we balanced the numbers of people, participants’ gender, and stimulation group across the three orders. The presentation order of the three different distributive contexts was also counter-balanced in the three orders among the participants receiving the three different stimulations. The trails were shown in fixed sequence to insure that the behaviors of the participants receiving different stimulations were completely comparable. However, within each pseudo-random order, the sequence of the contexts and the chips were counter-balanced with no obvious rules which may influence the expectations or behaviors of the participants (see Supplementary Material). These stories were presented one by one, and participants made distributive decisions by computers (**Figure 3**).

The participants were given 30 min to complete the task of income distribution. After the participants completed the distributive task, they were asked to complete a choice menu in 10 min and a questionnaire before finally receiving their payment.

We used the choice menu aims to provide a simple and direct measure of participants’ social preference of advantageous inequity aversion. The menu modified from Yang et al. (2016), consists of 10 decisions (cf. **Table 1**). In each, the decision maker (denoted by ‘proposer’) is asked to choose between two options (A and B). Each option allocates money to the proposer and to an anonymous other participant (denoted by ‘receiver’). For all options the payoff of the proposer is higher than for the receiver. This means that all cases yield advantageous inequality for the proposer, and it allows us to use this menu to measure participants’ advantageous inequity aversion parameter β (see Supplementary Material for detail). Each participant decides as if she is a proposer, because roles are not (randomly) determined until the end of the experiment.

**Table 1:** The choice menu of advantage inequity aversion.

Nr.	Option A	Option B	Choose B iff:
1	Yours: 370; Other’s: 180	Yours: 340; Other’s: 100	β ≤-0.60
2	Yours: 350; Other’s: 180	Yours: 340; Other’s: 100	β ≤-0.14
3	Yours: 330; Other’s: 180	Yours: 340; Other’s: 100	β ≤ 0.11
4	Yours: 310; Other’s: 180	Yours: 340; Other’s: 100	β ≤ 0.27
5	Yours: 290; Other’s: 180	Yours: 340; Other’s: 100	*β* ≤ 0.38
6	Yours: 270; Other’s: 180	Yours: 340; Other’s: 100	β ≤ 0.47
7	Yours: 250; Other’s: 180	Yours: 340; Other’s: 100	β ≤ 0.53
8	Yours: 230; Other’s: 180	Yours: 340; Other’s: 100	β ≤ 0.58
9	Yours: 210; Other’s: 180	Yours: 340; Other’s: 100	β ≤ 0.62
10	Yours: 190; Other’s: 180	Yours: 340; Other’s: 100	β ≤ 0.65

*The table presents the 10 decisions (given in rows) between options A and B. “Yours” refers to the proposer’s payoff and “Other’s” to the receiver’s payoff. The final column includes the values of β for which the inequity aversion model (1) rationalizes a choice of option A. Of course, this column was not shown to subjects*.

The payoffs for Option B remain constant across all 10 decisions, with the proposer earning 340, which is 240 more than the receiver (100). For the first decision, Option A gives the proposer more (370) and yields lower inequality (190) than B. Any non-negative β then implies higher utility for A than for B. Moving down along the table, the own earnings in Option A decrease, as does the inequality. This increases the level of advantageous inequity aversion needed to prefer Option A to B. The last column in the table gives these threshold values for β.

### Data Analysis

We first focused on comparing the distributive decisions of the participants across the different distributive contexts in the sham group, and we hoped for the result that the participants’ distributive decisions depended on the distributive context. To test the causal relationship between the activity of TPJ and participants’ distributive decisions, we investigated the distributive decisions across the three contexts in the different stimulation group. We further measured the participants’ social preferences of advantage inequity aversion in the different stimulation group and analyzed the correlation between the participants’ social preferences and their distributive decisions to the stratum of the lowest income to demonstrate the role of the participants’ social preferences in distributive decisions within the three distributive contexts.

Additionally, we used the participants’ distributive incomes to the three stratums and the Gini coefficient (a method measuring distributive fairness in economics) as data to represent the participants’ distributive decisions. The higher the value of Gene coefficient is, the more unfair our society is.

The Gini coefficient is usually defined mathematically based on the Lorenz curve, which plots the proportion of the total income of the population (y axis) that is cumulatively earned by the bottom x% of the population. An alternative approach would be to consider the Gini coefficient as half of the relative mean absolute difference, which is a mathematical equivalence (Sen, 1984). The mean absolute difference is the average absolute difference of all pairs of items of the population, and the relative mean absolute difference is the mean absolute difference divided by the average, to normalize for scale. If _x_i__ is the wealth or income of person *i*, and there are n persons, then the Gini coefficient _G_ is given by:

$$G=\frac{\Sigma_{i=1}^{n}\Sigma_{j=1}^{n}\left|x_{i}-x_{j}\right|}{2\;\Sigma_{i=1}^{n}\Sigma_{j=1}^{n}x_{j}}$$

Statistical analyses were performed using SPSS statistical software (version 20).

## Results

In the sham group, there was no significant difference in participants’ distributive incomes to stratum A (the highest income stratum) between the social-planner and veil of ignorance contexts (Mann–Whitney test: *z* = 0.478, *p* = 0.6326). However, the participants’ distributive incomes to stratum A in the context of social-planner and veil of ignorance contexts were both lower than that in the context of known position (Mann–Whitney test: *z* = -8.772, *p* < 0.01; *z* = -8.930, *p* < 0.01).

In the sham group, there was no significant difference in participants’ distributive incomes to stratum B (the midst income stratum) between the social-planner and veil of ignorance contexts (Mann–Whitney test: *z* = -0.102, *p* = 0.9187). However, the participants’ distributive incomes to stratum B in the social-planner and veil of ignorance contexts were both higher than that in the context of known position (Mann–Whitney test: *z* = 12.384, *p* < 0.01; *z* = 12.475, *p* < 0.01).

In the sham group, there was no significant difference in participants’ distributive incomes to stratum C (the lowest income stratum) between the social-planner and veil of ignorance contexts (Mann–Whitney test: *z* = -0.286, *p* = 0.7747). However, the participants’ distributive incomes to stratum C in the social-planner and veil of ignorance contexts were both higher than that in the context of known position (Mann–Whitney test: *z* = 13.065, *p* < 0.01; *z* = 13.002, *p* < 0.01). We have shown the mean and SD of distributive amounts across contexts and chips in the sham group (**Table 2**).

**Table 2:** The mean and SD of distributive amounts across contexts and chips in the sham group.

Chips	Contexts	The veil of ignorance			The social-planner			The known position		
	Stratums	A	B	C	A	B	C	A	B	C
30	Mean	12	10	8	12.8	10	7.2	22	4.8	3.2
	SD	4.08	0	4.08	4.58	0	4.58	9.13	5.10	4.76
60	Mean	26.8	19.2	14	24.4	20	15.6	45.2	9.2	5.6
	SD	7.48	2.77	6.45	5.07	0	5.07	14.47	8.62	7.12
90	Mean	36.4	29.6	24	36	29.6	24.4	56.4	19.2	14.4
	SD	7.57	2	7.07	9.13	2	8.21	20.99	11.87	10.03
120	Mean	51.2	39.6	29.2	48.4	39.2	32.4	84.4	21.6	14
	SD	9.71	2	8.62	11.79	4.93	9.26	28.59	17	12.91
150	Mean	60	48.8	41.2	60	50.4	39.6	110.4	22.4	17.2
	SD	12.25	3.32	10.13	11.18	4.55	11.36	38.24	21.27	17.68
180	Mean	71.2	58.4	50.4	69.2	59.6	51.2	131.2	27.2	21.6
	SD	13.64	3.74	10.98	13.52	2	12.36	47.20	25.90	21.92
210	Mean	82.4	68	59.6	81.2	68	60.8	147.2	36	26.8
	SD	17.39	5	13.38	15.09	5	11.15	49.88	28.43	22.68
240	Mean	97.6	80.4	62	92.8	79.2	68	170	40	30
	SD	15.08	4.55	15.55	18.82	6.40	15	58.24	32.91	26.46
270	Mean	106	88.4	75.6	107.2	88	74.8	198.8	40	31.2
	SD	16.33	5.54	14.17	26.85	8.66	21.63	70.79	39.48	32.32
300	Mean	117.2	98.4	84.4	115.6	99.6	84.8	221.2	43.6	35.2
	SD	21.51	4.73	18.73	23.47	4.55	20.64	78.97	43.67	36.41
Total	Mean	66.08	54.08	44.84	64.76	54.36	45.88	118.7	26.4	19.92
	SD	35.12	29.90	25.94	34.81	29.84	26.22	67.00	13.38	10.90

In addition, there was no significant difference in Gini coefficients for the distributions of income between the social-planner and veil of ignorance contexts (Mann–Whitney test: *z* = 1.225, *p* = 0.2204). However, the Gini coefficients for the distribution of income in the social-planner and veil of ignorance contexts were both lower than that in the context of known position (Mann–Whitney test: *z* = -15.891, *p* < 0.01; *z* = -16.202, *p* < 0.01). These results clearly indicated that the participants’ distributive decisions depended on the given context, and self-interest was an important factor in the distributions the participants knew their positions. The participants maximized their own payoff by increasing distributive income to the stratum they belong to and decreasing the distributive incomes to the other two stratums.

We then performed a two-way ANOVA for distributive proportions to stratum C with the stimulation type (right anodal/left cathodal tDCS, left anodal/right cathodal tDCS, sham stimulation) as a between-subject factor and the context (social-planner, veil of ignorance, known position) as a within-subject factor. There were significant main effects of stimulation type (*F*_(2,777)_ = 16.122, *p* < 0.01, $\eta_{p}^{2}$ = 0.014) and context (*F*_(2,777)_ = 608.555, *p* < 0.01, $\eta_{p}^{2}$ = 0.343). More importantly, a significant interactive effect of stimulation type and context was found (*F*_(4,775)_ = 11.398, *p* < 0.01, $\eta_{p}^{2}$ = 0.019). We further compared participants’ distributive proportions to stratum C among three stimulation types within different contexts. The participants’ distributive proportions to stratum C within the context of a known position after receiving right anodal/left cathodal tDCS and receiving right cathodal/left anodal tDCS over TPJ were both higher than after receiving the sham stimulation (*t*-test and Bonferroni corrections: right anodal/left cathodal tDCS, *p* < 0.01; right cathodal/left anoodal tDCS, *p* < 0.01). However, there was no significant difference between the two active stimulation groups (*t*-test and Bonferroni corrections: *p* = 0.997), and no significant difference among three stimulation types within the contexts of social planner and the veil of ignorance (*t*-test and Bonferroni corrections: social planner, right anodal/left cathodal tDCS vs. sham, *p* = 0.627, right cathodal/left anoodal tDCS vs. sham, *p* = 0.658; the veil of ignorance, right anodal/left cathodal tDCS vs. sham, *p* = 0.756, right cathodal/left anoodal tDCS vs. sham, *p* = 0.944). This finding indicates that the enhanced activity of the right TPJ (RTPJ) or the left TPJ (LTPJ) made the participants more averse to advantage inequity and made them more concerned about the distributive proportion to the lowest income stratum within the context of the known position.

In addition to the distributive proportion to the lowest income stratum, we also used the Gini coefficient to examine the participants’ equity-efficiency trade-offs in the income distribution. Two-way ANOVA on the Gini coefficient of income distribution was executed, with the context (social-planner, veil of ignorance, known position) as a within-subject factor and the stimulation type (right anodal/left cathodal tDCS, right cathodal/left anodal tDCS, sham stimulation) as a between-subject factor. We found a main effect of stimulation type (*F*_(2,777)_ = 3.525, *p* < 0.01, $\eta_{p}^{2}$ = 0.009) and of context (*F*_(2,777)_ = 797.401, *p* < 0.01, $\eta_{p}^{2}$ = 0.482). There was a significant stimulation type × context interaction (*F*_(4,775)_ = 30.160, *p* < 0.01, $\eta_{p}^{2}$ = 0.042).

To further evaluate the treatment effect, we also compared the Gini coefficients of participants’ distribution among three stimulation types within different contexts (**Table 3**).

**Table 3:** The mean and SD of Gini coefficient across conditions and stimulation types.

Context	R Anodal/L Cathodal		R Cathodal/L Anodal		Sham	
	Mean	*SD*	Mean	*SD*	Mean	*SD*
The veil of ignorance	0.2655	0.05958	0.2794	0.06489	0.2744	0.06443
The social planner	0.2641	0.04986	0.2631	0.04916	0.2708	0.06610
The known position	0.4232	0.1673	0.4025	0.1405	0.5181	0.1566

**FIGURE 4:**
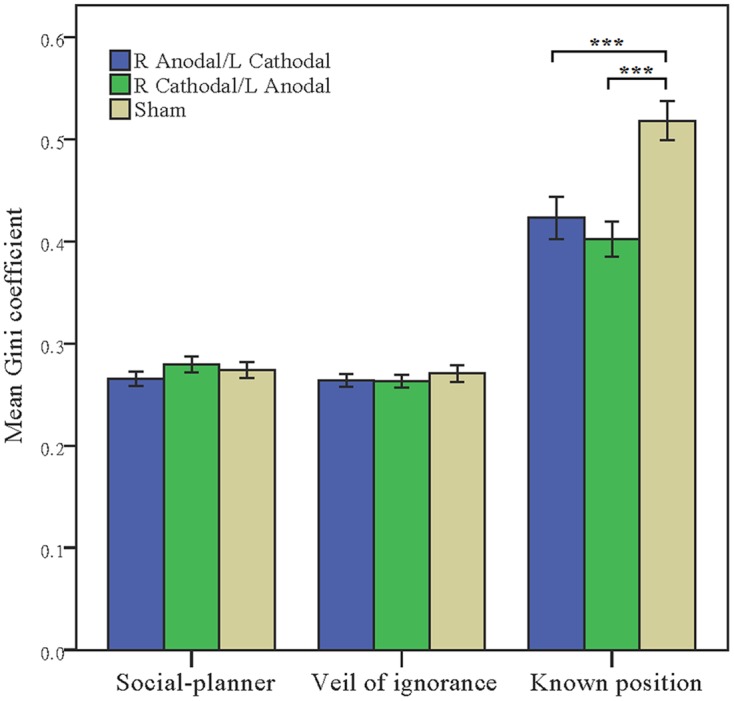
**Mean Gini coefficients of participants’ distribution across stimulation types over TPJ under the context of social-planner, veil of ignorance, known position**. Error bars indicate 95% confidence intervals. Asterisks indicate statistical significance of difference between treatments.

The Gini coefficients within the context of known position after receiving right anodal/left cathodal tDCS and receiving right cathodal/left anoodal tDCS over the TPJ were both lower than that after receiving the sham stimulation (*t*-test and Bonferroni corrections: right anodal/left cathodal tDCS, *p* < 0.01; right cathodal/left anoodal tDCS, *p* < 0.01) (**Figure 4**). However, there was no significant difference between the two active stimulation groups (*t*-test and Bonferroni corrections: *p* = 0.388), and no significant difference among three stimulation types within the contexts of social planner and the veil of ignorance (*t*-test and Bonferroni corrections: social planner, right anodal/left cathodal tDCS vs. sham, *p* = 0.323, right cathodal/left anoodal tDCS vs. sham, *p* = 1; the veil of ignorance, right anodal/left cathodal tDCS vs. sham, *p* = 0.515, right cathodal/left anoodal tDCS vs. sham, *p* = 0.352) (**Figure 4**). These results are fully consistent with the stimulation effect on the participants’ distributive proportions to stratum C.

**FIGURE 5:**
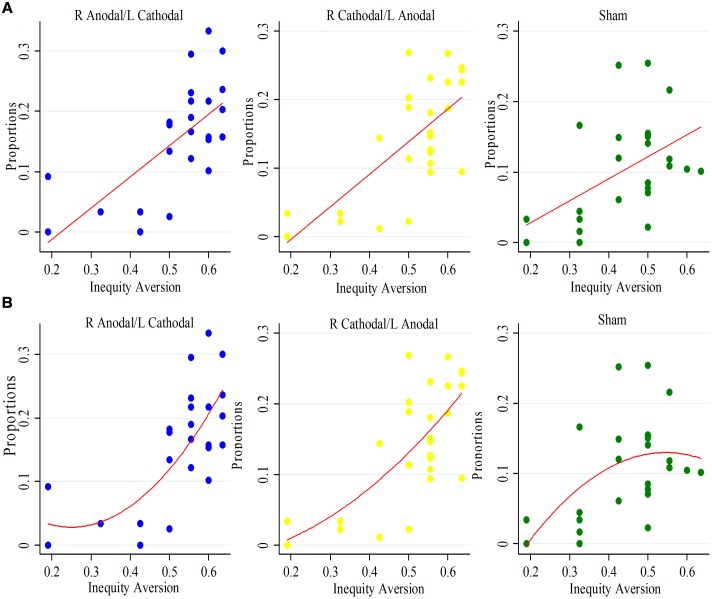
**Scatter plots of participants in the context of known position.** The horizontal axis represents the participants’ advantage inequity aversion and the vertical axis represents the distributive proportions to stratum C. **(A)** The line of best fits for scatter plots of participants receiving different stimulations. **(B)** The quadratic curve of best fits for scatter plots of participants receiving different stimulations.

We introduced participants’ social preferences about advantage inequity aversion to analyze the distributive decisions. We found that the participants’ distributive proportions to stratum C within the contexts of social planner and the veil of ignorance were not related to their social preferences about advantage inequity aversion (Spearman test: social planner, *p* = 0.2156; the veil of ignorance, *p* = 0.6499). However, the participants’ distributive proportions to stratum C within the context of known position was strongly related to their social preferences, which were measured with the choice menu (Spearman test: right anodal/left cathodal, *p* < 0.01; right cathodal/left anodal, *p* < 0.01; sham, *p* < 0.01). The result revealed that the participants who allocated more income to stratum C tended to exhibit more advantage inequity aversion in the choice menu and such an observation was robust in all of the three tDCS groups (**Figure 5A**). There is a steeper increasing trend of distributive proportions to stratum C with the increase of advantage inequity aversion in the line of best fit for right anodal/left cathodal and right cathodal/left anodal groups comparing to those for sham group (see **Figure 5** for scatter plots and line of best fits). The quadratic curve of best fits may indicate that the relationship between distributive proportions to stratum C and advantage inequity aversion seems tighter among participants with higher advantage inequity aversion in the right anodal/left cathodal and right cathodal/left anodal groups than the sham group (**Figure 5B**).

We also compared the participants’ social preference between the active stimulation and sham stimulation conditions. The advantage inequity aversion coefficient of the participants who received right anodal/left cathodal tDCS and received right cathodal/left anodal tDCS over the TPJ were higher than that of the participants who received the sham stimulation (Mann–Whitney test: right anodal/left cathodal tDCS, *z* = -2.442, *p* = 0.0146; right cathodal/left anodal tDCS, *z* = -2.496, *p* = 0.0126). These results indicated that tDCS to the TPJ altered the social preference about advantage inequity aversion of the participants (i.e., the participants were more generous and more concerned about the lowest stratum after receiving right anodal/left cathodal tDCS and receiving right cathodal/left anodal tDCS) and might led to relative changes in their distributive decisions under the context of known position.

## Discussion

Many previous studies from different fields have discussed the issues of income distribution justice and the factors that influence individuals’ distributive decisions (Friedman, 1953; Frohlich and Oppenheimer, 1992; Rutström and Williams, 2000). These studies focused on how people solved equity-efficiency trade-offs in income distribution (Harsanyi, 1955; Rawls, 1971; Dahlby, 1987). Such redistribution choices might be governed by self-concern (risk aversion) or social preference (inequality aversion) (Vickrey, 1945; Cowell and Kuga, 1981; Andreoni and Miller, 2002). However, evidence is lacking in the field of neuroscience regarding the link between distributive behavior and the regions of the brain that are possibly related to preference.

The present research complements these studies with tDCS by providing a causal relationship between distributive decisions across various social contexts and the activities of the TPJ. In addition to the brain stimulation results, we also investigated the participants’ behavioral data that was required to make distributive decisions in this distributive experiment to present a comprehensive theory about the role of social preference in distributive decisions in three different distributive contexts.

According to the behavioral data from the participants in the sham group across the three contexts, we found a context dependence of the distributive decisions, i.e., the participants distributive income to the highest income stratum in the known position context was significantly greater than those in the other two contexts, and the participants’ distributive income to the middle and lowest income stratum in the known position context was significantly lower than those in the other two contexts. Additionally, the Gini coefficients for the distribution incomes in the social-planner and veil of ignorance contexts were both lower than that of the context of known position. However, there was no significant difference in the participants’ distributive decisions (including the participants’ distributive income to the three income stratums and the Gini coefficients) between the contexts of social-planner and the veil of ignorance.

Consistent with prior distributive justice studies, these results demonstrated that the individuals displayed remarkable self-interest, and these decisions may be viewed as a posteriori rationalizations when the positions were known (Beckman et al., 2002). In the social-planner and the veil of ignorance contexts, the individuals tended to make more equal distributions among the three social stratums. The participants in the social-planner context decided on only the other participants’ payoffs without being paid themselves (Krawczyk, 2010). Hence, our experimental results revealed the existence of subjects’ self-interest in the context of known position and the equal income distribution in the contexts of social-planner and the veil of ignorance. Our results also demonstrated that the participants within the veil of ignorance preferred the distribution that maximized the well-being of the least well-off (Traub et al., 2009; Schildberg-Hörisch, 2010). However, the behavioral data were still unable to confirm whether participants’ equal distribution decisions in this context were the result of self-concern or impartial social preferences.

Based on the above behavioral results, we provided further neural evidence regarding distributive decisions in different contexts. First, there was no significant difference in the participants’ distributive proportions to the lowest income stratum among the three stimulation types within the contexts of social planner and the veil of ignorance. This finding indicates that after receiving tDCS over TPJ, the participants had not changed their equal distributive decisions within the contexts of social planner and the veil of ignorance. Second, we found that the participants allocated more income to the lowest income stratum within the known position after receiving right anodal/left cathodal tDCS and receiving right cathodal/left anodal tDCS over the TPJ. This finding reveals that enhanced in the activity of the right TPJ or left TPJ made the participants more averse to advantage inequity and made them more concerned about the distributive proportion to the lowest income stratum within the context of known position.

Many studies have shown that the activities of the RTPJ and the LTPJ are associated with the understanding of others’ mental states (Ciaramidaro et al., 2007; Sommer et al., 2007; Aichhorn et al., 2009; Ye et al., 2015b). Previous studies have demonstrated the involvement of the TPJ during decision-making in social preference by ultimatum game (Rilling et al., 2004; Declerck et al., 2013) and that gray matter (GM) volume in the TPJ is strongly associated with individuals’ altruism (Morishima et al., 2012). Specifically, Young et al. (2010) used TMS to the RTPJ to disrupt the capacity to understand others’ perspectives. Samson et al. (2004) reported evidence from brain-damaged patients that indicated that the patients with lesions in the LPTJ region exhibit impairment in false mental states tasks.

Additionally, relatively few significant cathodal-inhibition results have been revealed as compared to the anodal excitation effects according to prior tDCS studies. Such an asymmetric stimulation effect exists in cognitive or perceptual tasks. This issue has been deeply discussed by Jacobson et al. (2012), who argued that the lack of inhibitory cathodal effects might reflect compensation processes as cognitive functions are typically supported by rich brain networks. Hence, we inferred that the participants who received right anodal/left cathodal tDCS and right cathodal/left anodal tDCS over the TPJ exhibited an improvement in the capacity to understand others’ perspectives in altruistic behavior.

Together, the findings of these previous studies and our findings about the distributive decisions of participants receiving tDCS appear to indicate that the alterations of the social preferences of the participants after the receipt of tDCS to the TPJ might led to alterations in their distributive decisions within the context of known position. Specifically, the participants were likely to be more altruistic after receiving right anodal/left cathodal tDCS and after receiving right cathodal/left anodal tDCS over the TPJ, which made them more concerned about the distributive proportion to the lowest income stratum within the known position. However, the alterations of the social preferences of the participants after the receipt of tDCS to the TPJ did not lead to alterations in their distributive decisions within the contexts of social planner and the veil of ignorance.

To demonstrate our deductions, we provided more evidence about the stimulation effect on the Gini coefficient and advantage inequity aversion. A significant interactive effect of context and the stimulation type was found, and the Gini coefficients within the context of known position after receiving right anodal/left cathodal tDCS and receiving right cathodal/left anodal tDCS over the TPJ were both lower than after receiving the sham stimulation. However, there was no significant difference among the three stimulation types within the contexts of social planner and the veil of ignorance. These findings were fully consistent with the stimulation effect on the participants’ distributive proportions to the lowest income stratum across the different contexts. This finding indicates that the subjects’ social preference in the context of known position was changed after the receipt of tDCS to the TPJ and the equal income distributions in the contexts of social-planner and the veil of ignorance have not been changed by tDCS to the TPJ. Therefore, we might rule out the role of social preference in distributive decision within the contexts of social planner and the veil of ignorance.

We directly elicited participants’ advantage inequity aversion with a choice menu to further verify the role of social preference in distributive decisions across the different contexts. Powerful evidence indicated that the participants’ distributive proportions to the lowest income stratum within the known position were strongly related to the values of advantage inequity aversion. However, the participants’ distributive proportions to the lowest income stratum within the contexts of social planner and the veil of ignorance were not related to the values of advantage inequity aversion. These findings suggest that the participants’ income distribution within the known position depended on the degree of their advantage inequity aversion and the participants’ equal distributions within the contexts of social planner and the veil of ignorance were not derived from their altruistic social preference (advantage inequity aversion).

More importantly, we observed a significant stimulation effect on the advantage inequity aversion of the participants. The participants who received right anodal/left cathodal tDCS and who received right cathodal/left anodal tDCS over the TPJ were more averse to advantage inequity than the participants who received the sham stimulation. These findings are fully consistent with the stimulation effect on the participants’ distributive decisions within the known position. Therefore, the present study demonstrated that the modulation of the excitability of the TPJ might alter participants’ distributive decisions within the known position through the main driving force of these decisions, i.e., social preference about advantage inequity aversion.

However, it is obvious that participants’ distributive decisions within the contexts of social planner and the veil of ignorance have not been changed through the alteration of their advantage inequity aversion. When judged from within a veil of ignorance, income distributions are considered gambles (Friedman, 1953; Dahlby, 1987). In the context of social-planner, the social welfare function lacks any personal involvement (Brandt and Boulding, 1962; Cowell and Kuga, 1981; Lambert, 2001). Taken together, we can infer that participants’ equal distributions in the contexts of social planner and the veil of ignorance might be attributable to the social norm or risk attitudes, but not to the advantage inequity aversion.

In this study, we provided causal evidence regarding the function of the TPJ in income distributive decisions across various contexts and revealed that activation of this neural region can alter the participants’ distributive decisions within the known position, but no significant influence on the participants’ distributive decisions in the social planner and the veil of ignorance contexts were found. Our observations also indicate that participants’ social preference (advantage inequity aversion) is closely correlated with their distributive decisions under the known position; thus, the modulation of the activity of the TPJ might change participants’ distributive decisions within the known position by altering their social preferences, and the participants’ equal income distributions in the contexts of social planner and the veil of ignorance are not related to their social preference of altruistic behavior.

One limitation of the current study is that although our findings regarding the effect of stimulation over the TPJ on social preference were consistent with previous findings, the potential neural mechanism by which the specific brain area influences distributive decisions by altering social preference remains to be revealed and discussed. It cannot exclude that other psychological processes might be altered which influenced both the distributive decisions and social preferences while modulating TPJ through tDCS. Further brain imaging studies may focus on the dynamic activation of the TPJ while participants make distributive decisions across various contexts. Another option is to modulate the activities of other relative brain regions (e.g., DLPFC) to explore whether participants’ distributive decisions are changed, which would provide more information about intrinsic preferences related to distributive decisions across different contexts. Another deficiency of our study is that we cannot determine if the impact on distributive decisions and social preference are solely attributable to modulation of activity in the right TPJ or if the behavioral effects are the result of changing the balance of activity across both TPJs. Future studies may include neuroimaging measures to explore the neural changes associated with neuromodulation leading to behavioral effects and also to explore other paradigms of stimulation, such as unilateral stimulation. Moreover, the between-subject design in the current study highly relied on the hypothesis that the original levels of participants’ advantageous inequity aversion were identical among the three stimulation groups. The correlations between advantageous inequity aversion and distributive justice under the three contexts are not completely valid. These issues caused by heterogeneity should be considered seriously in our future studies which may be improved by more precise designs such as applying a within-subject design or performing a pre-test inequity aversion level measurement. No significant difference between the social-planner and veil of ignorance contexts may due to misconception about the experiment paradigm in some participants which should also be improved in further experimental designs.

## Conclusion

To conclude, our experiment demonstrated that modulating the excitability of the TPJ using tDCS altered the distributive decisions of the participants under the known position, and this effect might be attributable to a change in the individuals’ social preferences. We also found that participants’ distributive decisions under the contexts of social planner and the veil of ignorance have not been changed through the alteration of their advantage inequity aversion. It suggests that the participants’ equal income distributions in the contexts of social planner and the veil of ignorance are not related with their social preference of altruistic behavior.

## Author Contributions

JL, SC, DH, HY, HZ designed experiment; JL, SC, DH, HY, HZ performed experiment; JL analyzed data; JL drew figures; JL, SC, DH, HY, HZ wrote the manuscript; JL, SC, DH, HY, HZ revised the manuscript and JL, SC, DH, HY, HZ finally approved the version to be published.

## Conflict of Interest Statement

The authors declare that the research was conducted in the absence of any commercial or financial relationships that could be construed as a potential conflict of interest.
