# Supplementary material for: Whether Modulating the Activity of the Temporalparietal Junction Alters Distribution Decisions within Different Contexts: Evidence from a tDCS Study
Source: Front Psychol. 2017 Feb 21;8:224. doi: 10.3389/fpsyg.2017.00224 (PMC5318427; doi:10.3389/fpsyg.2017.00224)
Supplement: Supplementary file 1 [file Data_Sheet_1.DOCX]

(1)Estimation Algorithms for *β*:

Using Subject *j*’s decisions in the choice menu, we estimate her parameters *β_j_* in the following three consecutive steps. Before we present these steps, it is useful to define the values listed in the last column of the menu for decision *k* as *v_k_.* To do so formally, denote by *o_kA_* = (*x_kA_*, *y_kA_*), *o_kB_* = (*x_kB_*, *y_kB_*) the payoff pairs to individuals *x* (the decision maker) and *y* in decision *k* (=1,…10) if option A or B, respectively, is chosen. Then, for the menu we have:

*v_k_*$\equiv{min}_{\beta:U(O_{kA})\geq U(O_{kB})}\beta$

Step 1: Define the ‘switching point’, *σ_j_*, as the first decision for which the Subject *j* chooses option B in the menu. If *j* always chooses A, her switching point equals 11. Hence *σ_j_*$\in${1,…11}. If *j* chooses A at some point in the menu after choosing B earlier in the menu, define *σ_j_* = –∞.

Step 2: All *j* for whom $\exists$*i*, such that *σ_j_* = –∞ are categorized as ‘inconsistent’, implying that no *β* exist that allow her choices to be rationalized by the inequity aversion model. Denote the set of ‘consistent’ subjects by C (={*j*| *σ_j_* > 0, $\forall$*i*}).

Step 3: Let *j*$\in$C,

Any *σ_ij_* $\in$ {2,…,10} can be rationalized by a finite range of parameter values in the model. In particular, an upper bound for the relevant parameter (*β*) is given by *v_ik_*.

This is denoted by $\bar{\tilde{\beta}}$*_j_* = *v*_σ_*_j_*. A lower bound of the model estimate is given by $\underline{\tilde{\beta}}$*_j_* = *v*_σ_*_j-_*_1_. As our estimate of the advantage inequity aversion parameter for Subject *j* we choose the average of the upper and lower bounds: β*_j_* = (${\bar{\tilde{\beta}}}_{j}+\underline{\tilde{\beta}}$*_j_*)/2 _,_ respectively.

If *σ_j_=*1, we define *v*_σ_*_j-_*_1_ = −∞. *v*_σ_*_j_* still determines the upper bound for the model estimates, $\bar{\tilde{\beta}}$*_j_*. Because the lower bound $\underline{\tilde{\beta}}$*_j_* is –∞, we cannot take the mean as an estimate. Instead, we choose the upper bound as the model estimate in this case, i.e., β*_j_* = $\underline{\tilde{\beta}}$*_j_*.

Finally, if *σ_j_=*11, then we still have lower bound $\underline{\tilde{\beta}}$*_j_* = *v*_σ_*_j-_*_1_ = *v*_10_, but the upper bounds ${\bar{\tilde{\beta}}}_{j}$= +∞. Hence, we choose the value of the lower bound as the model estimate in this case, that is, β*_j_* = $\underline{\tilde{\beta}}$*_j_*.

(2)Pseudo-random order Sample:

| Order | Context | chip |
| --- | --- | --- |
| 1 | veil of ignorance | 150 |
| 2 | social-planner | 240 |
| 3 | known position | 90 |
| 4 | veil of ignorance | 60 |
| 5 | social-planner | 120 |
| 6 | known position | 210 |
| 7 | veil of ignorance | 30 |
| 8 | social-planner | 270 |
| 9 | known position | 240 |
| 10 | social-planner | 30 |
| 11 | known position | 120 |
| 12 | veil of ignorance | 180 |
| 13 | veil of ignorance | 210 |
| 14 | social-planner | 90 |
| 15 | social-planner | 210 |
| 16 | known position | 180 |
| 17 | known position | 150 |
| 18 | veil of ignorance | 90 |
| 19 | known position | 60 |
| 20 | social-planner | 150 |
| 21 | social-planner | 180 |
| 22 | veil of ignorance | 300 |
| 23 | veil of ignorance | 240 |
| 24 | known position | 30 |
| 25 | known position | 270 |
| 26 | social-planner | 300 |
| 27 | veil of ignorance | 120 |
| 28 | veil of ignorance | 270 |
| 29 | known position | 300 |
| 30 | social-planner | 60 |
